# The Role of Pneumococcal Virulence Factors in Ocular Infectious Diseases

**DOI:** 10.1155/2018/2525173

**Published:** 2018-11-13

**Authors:** Angela H. Benton, Mary E. Marquart

**Affiliations:** Department of Microbiology and Immunology, University of Mississippi Medical Center, Jackson, MS 39216, USA

## Abstract

*Streptococcus pneumoniae* is a gram-positive, facultatively anaerobic pathogen that can cause severe infections such as pneumonia, meningitis, septicemia, and middle ear infections. It is also one of the top pathogens contributing to bacterial keratitis and conjunctivitis. Though two pneumococcal vaccines exist for the prevention of nonocular diseases, they do little to fully prevent ocular infections. This pathogen has several virulence factors that wreak havoc on the conjunctiva, cornea, and intraocular system. Polysaccharide capsule aids in the evasion of host complement system. Pneumolysin (PLY) is a cholesterol-dependent cytolysin that acts as pore-forming toxin. Neuraminidases assist in adherence and colonization by exposing cell surface receptors to the pneumococcus. Zinc metalloproteinases contribute to evasion of the immune system and disease severity. The main purpose of this review is to consolidate the multiple studies that have been conducted on several pneumococcal virulence factors and the role each plays in conjunctivitis, keratitis, and endophthalmitis.

## 1. Introduction

*Streptococcus pneumoniae* (*S. pneumoniae*) is a gram-positive, facultatively anaerobic pathogen responsible for many severe infections in different body sites [1].* S. pneumoniae* frequently colonizes the nasopharynx of healthy adults [1, 2]. While many healthy adults asymptomatically carry this bacterium, it is a leading cause of severe diseases such as pneumonia, meningitis, septicemia, and middle ear infections [3, 4].* S. pneumoniae* continues to be one of the main causes for infectious diseases of the ocular surface such as keratitis and conjunctivitis, along with coagulase-negative* Staphylococcus*,* Staphylococcus aureus, *and* Pseudomonas aeruginosa *[5–9]. The following review will cover (A) three pneumococcal ocular infectious diseases: conjunctivitis, keratitis, and endophthalmitis and (B) the role specific pneumococcal virulence factors play in pathogenesis during each infection.

*S. pneumoniae *has many virulence factors including a polysaccharide capsule, pneumolysin, neuraminidases, and zinc metalloproteinases, all of which contribute to the severity of ocular infections [10]. The pneumococcal capsule aids in the evasion of the host complement system by reducing both IgG and C-reactive protein binding [10–12]. Since* S. pneumoniae* avoids activating the complement system, it is less likely to be phagocytosed by neutrophils [13]. Both pneumococcal vaccines currently approved for use cover the most common pneumococcal serotypes involved in pneumonia and invasive diseases by targeting the capsule [14]; however, nonencapsulated* S. pneumoniae *(NESp) are the cause of most cases of conjunctivitis [15, 16]. There are two classification of NESp. Group I has the capsule polysaccharide biosynthetic (*cps*) locus but does not produce capsule due to a mutation or deletion [17, 18]. Group II does not have these cps genes but instead has novel oligopeptide binding proteins* aliC*,* aliD*, and/or the putative adhesin* pspK *[17, 19, 20]. Conjunctivitis strains have been identified as belonging to a subset clade of Group II which harbor* aliC* and* aliD* but not* pspK *[19].

When grown to stationary phase* in vitro*,* S. pneumoniae* spontaneously undergo self-lysis [21–23]. LytA, the main autolysin of S*. pneumoniae*, is indicated as an important virulence factor in several disease models [24–27]. There are 3 hypotheses for the mechanism behind the contribution of LytA to pneumococcal virulence. One theory suggests LytA is released by competent pneumococcal cells to lyse noncompetent pneumococcal cells in the same environment [28]. This would allow for easy DNA exchange and integration by the naturally competent cells. A second hypothesis proposed that autolysis is induced to interfere with the cascade of host mediated immune responses [22, 29]. Phagocytosis of intact pneumococcal as well as phagocyte-stimulating cytokines is significantly reduced by autolyzed pneumococci [29]. Another suggestion for pneumococcal self-lysis is that lysis is induced to release other virulence factors, such pneumolysin [30]. Pneumolysin (PLY) is one of the most widely studied pneumococcal virulence factors. It has a damaging effect in every type of ocular infection covered in this review [31–38]. PLY is a cholesterol-dependent cytolysin that functions as a pore-forming toxin [38, 39]. This family of cytolysins also includes perfringolysin, streptolysin, and listeriolysin [39]. In addition to its cytolytic activity, PLY binds the Fc region of antibodies, which leads to a cascade of host mediated inflammation by activating the classical complement pathway [11, 40]. In some cases, PLY alone can cause as much damage alone as the bacteria producing it due to the massive immune response from the host.

In order to cause systemic disease, pneumococcus must first be able to colonize the nasopharynx [20, 41].* S. pneumoniae *produces 3 neuraminidases (Nan), NanA, NanB, and NanC that assist in adherence and colonization [42–44]. Both NanA and NanB function as sialidases, exposing cell surface receptors to pneumococcus [10, 42]. Adherence and colonization are less likely to happen without the appropriate cell surface receptors being exposed by the neuraminidases; thus, disease states are also less likely to be established. Once a systemic infection is established,* S. pneumoniae* can become an invasive disease due to hyaluronate lyase [10]. Hyaluronate lyase belongs to a family of enzymes known as hyaluronidases [45]. These enzymes function as a virulence factors by breaking down the extracellular matrix components and increasing tissue permeability [46].* S. pneumoniae* hyaluronate lyase cleaves the 1,4-glycosidic linkage in hyaluronan between the N-acetyl-*β*-d-glucosamine and d-glucuronic acid residues [47]. Hyaluronan has been found as component of the extra cellular matrix in every tissue and fluid of both humans and animals [10, 45]. Cleavage of hyaluronan during an infection implicates hyaluronate lyase as a potential pneumococcal virulence factor that promotes tissue invasion.

*S. pneumoniae* produces three zinc metalloproteinases (Zmp), IgA1 protease, ZmpB, and ZmpC [48]. IgA1 protease cleaves neutralizing IgA1 antibodies at the hinge region [49]. Also, the pneumococcal production of IgA1 protease is necessary for bacterial adherence to epithelial cells [50]. ZmpB has been found in every isolated strain of* S. pneumoniae *[51, 52]. ZmpB causes an increase in tumor necrosis factor-alpha (TNF-*α*) concentration, which can exacerbate the severity of pneumococcal pneumonia and septicemia [53]. ZmpC can cleave host metalloproteinase-9 (MMP-9) and cause the removal of mucins from epithelial cells [54, 55]. Additionally, ZmpC binds to P-selectin and effectively inhibits neutrophil migration [56], as the binding of P-selectin to PSGL-1 neutrophil migration [57]. These zinc metalloproteinases not only contribute to evasion of the immune system but also the progression of serious pneumococcal diseases.

## 2. Infectious Diseases

### 2.1. Conjunctivitis

Approximately 1.35% of conjunctival infections are caused by bacteria [58], while allergens and viruses are more common culprits [59]. Even though bacterial conjunctival infections are less common, the indirect and direct costs of treatment is estimated to total over $500,000,000 in the United States [58]. Typical infections are associated with redness, edema, purulent discharge [60], and occasionally light sensitivity [6, 61]. The most common bacterial pathogens isolated in adult conjunctival infections are the staphylococcal species [62]; however, conjunctivitis in children is more often caused by* Haemophilus influenzae, S. pneumoniae, *and* Moraxella catarrhalis *[59, 62].* Neisseria gonorrhoeae *is commonly the isolated pathogen in cases of hyperacute bacterial conjunctivitis, which presents with swelling of the eyelid, pain, and purulent discharge [59, 63]. Pneumococcal conjunctival infections can be caused by poor contact lens hygiene, contaminated cosmetics, or living in close contact with others (military barracks, college dormitories, etc.) [16, 64]. While these infections are painful, they are treatable with topical antibiotics [65]. Patients receiving proper treatment for bacterial conjunctivitis recover with little to no vision loss [66].

The pneumococcal capsule, a well-studied virulence factor, is unnecessary for infection in the rabbit conjunctivitis model [7]. While both encapsulated and NESp have been isolated from patients with conjunctival infections, it is more often caused by the nontypeable strains of pneumococcus [7, 15, 16, 64, 67]. In a 2014 study, Valentino* et al.* collected 271* S. pneumoniae* conjunctivitis isolates during clinical treatment. They determined through multilocus sequence typing that over 90% were nonencapsulated [15]. It is important to note that several NESp isolates have been isolated during conjunctival outbreaks [20, 64, 68]. Until recently it was thought that NESp was unlikely to cause diseases other than conjunctivitis [20, 69]. A recent publication by Bradshaw* et al.* showed the novel NESp oligopeptide* aliD* to be instrumental for production of cytolytic levels of pneumolysin in a virulent strain (MNZ41) [70]. The same study shows adhesion and colonization to be significantly higher in NESp strains containing both* aliC *and* aliD *[70]. This recent study by Bradshaw* et al.* clearly show the potential NESp has to cause disease, especially NESp that contain* aliC* and* aliD* such as the epidemic conjunctivitis strains of NESp [15].

Though capsule is not necessary, pneumococcal neuraminidase activity increases in the absence of capsule during conjunctivitis [7, 52]. In fact, at 3 and 12 hours after infection a capsule-deficient mutant exhibited significantly more neuraminidase activity than the parent strain in a rabbit conjunctivitis model [7]. Also, nonencapsulated pneumococcal conjunctivitis isolates produce significantly higher neuraminidase activity after 6 hours of bacterial exposure to higher mucin-expressing corneal epithelial cells [68].

Conjunctivitis strains of NESp, as well as encapsulated strains, also secrete a zinc metalloproteinase (ZmpC) that causes enhanced bacterial internalization by removing of specific mucins from the epithelium [55]. Mucins are proteins, and depending on their molecular structure, can be either secreted or membrane-associated [71, 72]. Mucin 16 (MUC16) is part of the ocular surface glycocalyx and is also thought to provide a barrier to the epithelial surface [73, 74]. The removal of MUC16 by ZmpC, allows the pneumococcus to adhere and subsequently invade human conjunctival epithelial cells significantly better than the same strain lacking ZmpC [55]. ZmpC also cleaves human matrix metalloproteinase 9 (MMP-9), which is a key player in wound repair of epithelium [54, 75]. MMP-9 is upregulated in the eye by inflammatory cytokines TNF-*α* and interleukin-1-beta (IL1*β*) during a disease state or microbial infection [76–78]. With the ability to remove MUC16 from the conjunctival epithelium and cleave MMP-9 [55], an infection with a ZmpC-producing strain increases the chance of a more severe pneumococcal infection.

### 2.2. Keratitis

Keratitis caused by pneumococcus can lead to corneal scarring, which can result in permanent visual reduction [66]. Infections are commonly caused by improper contact lens wear, trauma, or previous ocular surgery [79–81].* S. pneumoniae* has been identified as one of the major agents of bacterial keratitis along with* Staphylococcus aureus* and* Pseudomonas aeruginosa* [82–84]. An analysis of bacterial keratitis cases over a 5-year span in one hospital showed* S. pneumoniae *was responsible for 38% of infections,* P. aeruginosa *for 29%, and* S. aureus *for 4% [85]. Other analyses of etiologic agents causing bacterial keratitis indicate similar distribution among pathogens [86, 87]. Improper keratitis treatment can lead to corneal ulcers [7, 66]. This ulceration of the cornea can result in a penetrating wound and lead to endophthalmitis [88]. Current treatment of bacterial keratitis consists of topical broad spectrum antibiotics [87, 89]; however, antibiotics alone may not be the most effective treatment since the pathogenesis during pneumococcal keratitis is not from bacterial burden alone [90].

The polysaccharide capsule of* S. pneumoniae* and PLY have been investigated for their roles in the progression of keratitis [7, 9, 34, 35]. Using the rabbit keratitis model, one study compared bacterial burden and infectivity of Avery's strain, a well-characterized encapsulated strain, and R6, the nonencapsulated strain [9]. Reed and colleagues showed no significant difference at 20 hours in bacterial burden or disease severity based on biomicroscopy examination. At 48 hours after infection, they recovered significantly more bacteria from corneas inoculated with Avery's encapsulated strain than with the nonencapsulated strain. The rabbits cleared the nonencapsulated strain more quickly than the encapsulated strain, but not before the host mediated inflammatory reactions caused damage to the corneas. This finding indicated that capsule was not necessary for pathogenesis as there was no significance difference in the biomicroscopy scores.

The host reaction to PLY causes much of the inflammation observed during pneumococcal keratitis [38]. Studies with strains lacking PLY have shown reduced virulence when compared to the parent strain in the rabbit keratitis model [31, 35, 38]. Pneumolysin appears to perform its dual roles of cytolytic activity and elicitation of inflammation during keratitis. First, PLY binds to lipid rafts in the corneal epithelial cell membrane prior to subunit oligomerization and pore formation, resulting in host cell lysis [34]. Secondly, PLY elicits increased infiltration of neutrophils into the cornea as evidenced by histopathology of corneas infected with wild type bacteria compared to PLY-deficient bacteria [32, 91]. These findings indicated that much of the damage caused during keratitis likely results from both direct corneal cell death by PLY and immune-derived damage from proinflammatory signaling in response to PLY. While capsule does not seem to play a significant role in the progression of keratitis, PLY is a key virulence factor in the devastation caused to the corneal cells both* in vivo* and* in vitro* [9, 31, 32].

### 2.3. Endophthalmitis

Though keratitis and conjunctivitis have a higher incidence than endophthalmitis, these infections are usually much less severe and easier to treat and carry a lower risk of vision loss or enucleation [62, 63, 66, 82, 92–95]. Approximately 0.05% of patients undergoing intraocular surgery develop bacterial endophthalmitis, resulting in a relatively low incidence of disease [96, 97]. The infection most commonly occurs after cataract removal, intravitreal injections, or a penetrating eye trauma [96–100]. The three main pathogens that cause bacterial endophthalmitis are coagulase-negative Staphylococcus (70%),* Staphylococcus aureus *(10%), and streptococcal species (9%) [100–103]. But, streptococcal species were three times as likely to be the cause of bacterial endophthalmitis in patients receiving intravitreal injections from ophthalmologists who did not wear facial masks [104].

The polysaccharide capsule is necessary for full virulence in pneumococcal endophthalmitis [61, 105]. In a study comparing a capsule-deficient isogenic mutant of a* S. pneumoniae* clinical isolate to the parent strain in a rabbit endophthalmitis model, both animal groups suffered from vitreal infections [105]. However, rabbits infected with parent strain exhibited significantly higher biomicroscopy scores at 24 and 48 hours, indicating a more severe disease [105]. The same study showed significantly more bacteria were recovered from the eyes infected with the parent strain as well. In a separate study, the same group investigated the benefits of passive immunization with Pneumovax®23 (one of the currently approved pneumococcal capsule-based vaccines) to prevent infection due to prevalence of encapsulated pneumococcus causing endophthalmitis. While the results did show less severe symptoms and lower biomicroscopy scores for rabbits immunized with Pneumovax®23, bacteria were still able to grow and subsequently cause disease [36].

Similarly, an intraocular infection with a PLY-deficient strain also resulted in less tissue damage and lower biomicroscopy scores [25, 106]. In a study comparing eye infections with strains that produce different amounts of pneumococcal toxin, strains with higher PLY activity caused more inflammation and tissue damage [107]. Interestingly, PLY by itself in the vitreous humor can cause the same tissue damage and histopathology seen in an infection with the bacteria [106]. A 2010 study by Sanders* et al*. immunized rabbits with antiserum to PLY as an attempt to prevent damage accrued by the cytolytic toxin. The immunized rabbits had significantly lower biomicroscopy score at 24 and 48 hours, and less retinal damage; however, there was not a significant difference in bacterial burden recovered from the vitreous [37].

The deletion of neuraminidase genes, on the other hand, has shown a very different effect during endophthalmitis. NanA deficient, NanB deficient, and NanAB deficient strains were tested in the rabbit endophthalmitis model [108]. The loss of the neuraminidases did not significantly decrease the severity of disease, but rather eyes infected with the mutants had significantly higher biomicroscopy scores indicating a more severe disease [108].

Aggressive treatments for streptococcal endophthalmitis infections include removal of the vitreous humor (vitrectomy) and intravitreal injections of combinations of antibiotics including vancomycin and either ceftazidime or amikacin [99, 109]. Patients receiving intraocular injections of vancomycin unfortunately are at risk for developing hemorrhagic occlusive retinal vasculitis (HORV), which can also lead to significantly decreased visual acuity or enucleation [110]. HORV is typically caused by a delayed onset hypersensitivity to vancomycin, occurring approximately 8 days after administration [110]. Topical antibiotic drops also have no prophylactic effect on bacterial endophthalmitis [111]. Both disease and treatment cause corrected visual acuity outcomes of 20/200 to 20/70 in affected eyes; therefore, the need to develop better endophthalmitis therapies is vital [110, 111].

## 3. Future Perspectives

Researchers have extensively studied many of the pneumococcal virulence factors in systemic disease models, but we know far less about the impact in the ocular environment, as outlined in Table 1.

For instance, a literature search for PLY knockout studies in a conjunctivitis model yields no results. Nonencapsulated strains are the predominant cause of pneumococcal conjunctivitis, which indicates that one or more factors other than capsule is involved during infection, and PLY is a prime candidate given its involvement in keratitis and endophthalmitis [7, 15, 16, 64]. Therefore, without PLY to initiate an inflammatory cytokine cascade [32, 106, 113], the damage is likely to be less severe when compared to the parent strain. As seen in a keratitis study, topically applied cholesterol negates much of the damage caused by PLY, due to cholesterol's ability to inactivate PLY [38]. The same treatment approach could be investigated for conjunctivitis to spare overuse of antibiotics.

*S. pneumoniae* also has three other zinc metalloproteinases, IgA1, ZmpB, and ZmpD [48, 114]; yet, at the time this review was written, none have been studied in the context of ocular diseases. ZmpB induces a TNF-*α* inflammatory response, similar to PLY [113], when* S. pneumoniae* infects the lower respiratory tract of mice [53]. TNF-*α* not only changes the morphology, but also damages the cytoplasm of rabbit corneal cells [115]. Mice infected intranasally with a strain lacking ZmpB had significantly lower cytokine levels than mice infected with the wild type strain [53]. Therefore, it is possible that ZmpB might play a major role in both keratitis and endophthalmitis by initiating the host inflammatory cascade.

In conjunctivitis and keratitis, the presence of pneumococcal neuraminidases leads to more severe disease [7, 15]; the opposite is true in the intraocular environment. Only one study has analyzed the pathogenicity potential of neuraminidases in endophthalmitis [108]. Increased expression of* nanA* and* nanB* were seen during pneumococcal endophthalmitis; however, a deletion of* nanA*,* nanB*, or both caused a significant increase in biomicroscopy scores [108]. Interestingly, capsule gene expression was also decreased [108] even though the capsule is required for full virulence during endophthalmitis [105]. One possibility of explaining these findings is that neuraminidase activity and capsule expression are coordinately regulated and deletion of one or the other results in differential pathogenic effects. For conjunctivitis, deletion of capsule results in increased neuraminidase production [7]. For endophthalmitis, deletion of neuraminidase and the resulting decrease in capsule expression might involve an alternate mechanism of regulation with a different effect than that from complete deletion of the capsule locus.

Another aspect of pneumococcal ocular infections that we know very little about is impact of the available nutrients. The intraocular environment provides an ideal niche for bacterial growth.* S. pneumoniae *has been shown to proliferate to 10^9^ CFU/mL in rabbit vitreous [105], indicating an abundance of nutrients. The nutrients in the vitreous humor as well as the metabolic mechanisms that allow* S. pneumoniae *to propagate to such high numbers in the ocular environment remain unknown.* S. pneumoniae *is able to metabolize over 30 carbon sources* in vitro *[116], but prefers glucose as a carbon source [117–120]. Glucose is readily available in the intraocular environment, and in concentrations that mimic those found in blood [121]. However, one study showed that an abundance of glucose made no significant difference on the number of bacteria recovered from an endophthalmitis infection by utilizing insulin dependent diabetic rabbits [122]. Future investigations should focus on resolving this discrepancy.

## 4. Conclusion

While* S. pneumoniae* remains one of the leading causes of serious systemic diseases such as bacterial pneumonia and meningitis, it is also a major cause of concern in the realm of ocular infections.* S. pneumoniae* remains one of the top causative bacterial pathogens for all three types of ocular infections described in this review. Conjunctivitis is treatable with topical antibiotics, keratitis is treatable but can lead to corneal scarring, and endophthalmitis more often than not leads to severe vision loss and possible enucleation. Though two pneumococcal vaccines exist for the prevention of nonocular diseases, they do little to fully prevent ocular infections.

This pathogen has several virulence factors that wreak havoc on the conjunctiva, cornea, and intraocular system. The polysaccharide capsule allows the bacterium to evade the complement system. PLY mediates an inflammatory cascade that can be just as damaging, if not more so, than the bacterium itself.* S. pneumoniae* also possess three neuraminidases (NanA, NanB, and NanAB) that play a role adhesion and subsequently colonization. The metalloproteinase ZmpC removes crucial glycoproteins that are necessary for the recruitment of MMP-9, an essential metalloprotease for wound healing.

A better picture of pneumococcal virulence factors in previously unexplored ocular infection types would lead to a broader knowledge of their roles in pathogenesis. Understanding the nutritional landscape of the intraocular environment and pneumococcal metabolism could reveal novel virulence mechanisms. Most importantly, more knowledge about how the pneumococcus causes ocular infections and damage to the eye could potentially lead to sight-saving drug discoveries. Development of new therapeutics is especially important for endophthalmitis because one of the most used treatment options can, unfortunately, lead to ocular damage and loss of sight.

## Figures and Tables

**Table 1:** Virulence factor requirement during different ocular infectious diseases.

**Virulence factor**	**Conjunctivitis**	**Keratitis**	**Endophthalmitis**
	(reference)	(reference)	(reference)
**Polysaccharide capsule**	dispensable [7, 15, 64, 68, 112]	dispensable [7, 9]	necessary [105]

**Pneumolysin (PLY)**	Unknown	necessary [9, 32, 34]	necessary [25, 36, 37, 106]

**Neuraminidase**	necessary [7, 68]	necessary [7, 61]	unknown [108]

**Zinc metalloproteinase C (ZmpC)**	necessary [54, 55]	necessary [55]	unknown
